# Emerging concepts in atopic dermatitis and atopic keratoconjunctivitis

**DOI:** 10.1097/ACI.0000000000001102

**Published:** 2025-08-06

**Authors:** Keya Jafari, Virginia L. Calder

**Affiliations:** a UCL Institute of Ophthalmology; b Biomedical Research Centre, UCL & Moorfields Eye Hospital, London, UK

**Keywords:** atopic dermatitis, atopic keratoconjunctivitis, biologics, dupilumab, interleukin-4/13 receptor

## Abstract

**Purpose of review:**

Atopic dermatitis (AD) is a chronic inflammatory skin disease involving Th2 cytokine-driven inflammation. AD patients are at risk of developing conjunctivitis, including atopic keratoconjunctivitis (AKC), a sight-threatening chronic allergic eye disease involving both Th2 and Th1/17 cytokine-driven inflammation.

In AD, dupilumab is highly effective by inhibiting interleukin (IL)-4 and IL-13. However, some AD patients develop dupilumab-associated ocular surface disease (DAOSD), an AKC-like disease. There are no biomarkers to predict who will develop DAOSD. The purpose of this review is to highlight recent findings in AD and AKC suggesting different immunopathogenic mechanisms are involved.

**Recent findings:**

A recent proteomics study of tear fluids identified raised inflammatory markers (*n* = 31) in AD patients with DAOSD (*n* = 22) and a shift towards a Th1/17 profile. Alternative biologics have been investigated for treating moderate-to-severe AD which have fewer ocular side-effects. The inhibitory effects of dupilumab cause an associated upregulation of IL-33 which could lead to an AKC-like disease. A recent therapeutic approach in AD via regulatory T cells suggests a novel treatment for those at risk of DAOSD.

**Summary:**

Ocular side-effects of dupilumab suggest that the immunopathogenic pathways in moderate-to-severe AD and AKC are not the same and, for DAOSD, might require different treatment approaches.

## INTRODUCTION

Whereas atopic dermatitis (AD) is a well established, polarized Th2-driven disease, the evidence for Th2-mediated inflammation in atopic keratoconjunctivitis (AKC) is less clear. Increases in both Th1 and Th2 AKC conjunctival CD4-T cells were first reported decades ago [1] and it was initially thought that conjunctival Th2 cells were driving the excessive tearing, whereas the Th1 cells were driving an epithelial pathology. The well established functional antagonism between interleukin (IL)-4, IL-17 and interferon gamma (IFNγ) suggested there was a fine balance between Th2, Th17 and Th1 cells. In the absence of an *in vivo* model for AKC, the CD4-T cell subset responsible for promoting chronic conjunctival inflammatory disease remains unknown. 

**Box 1:**
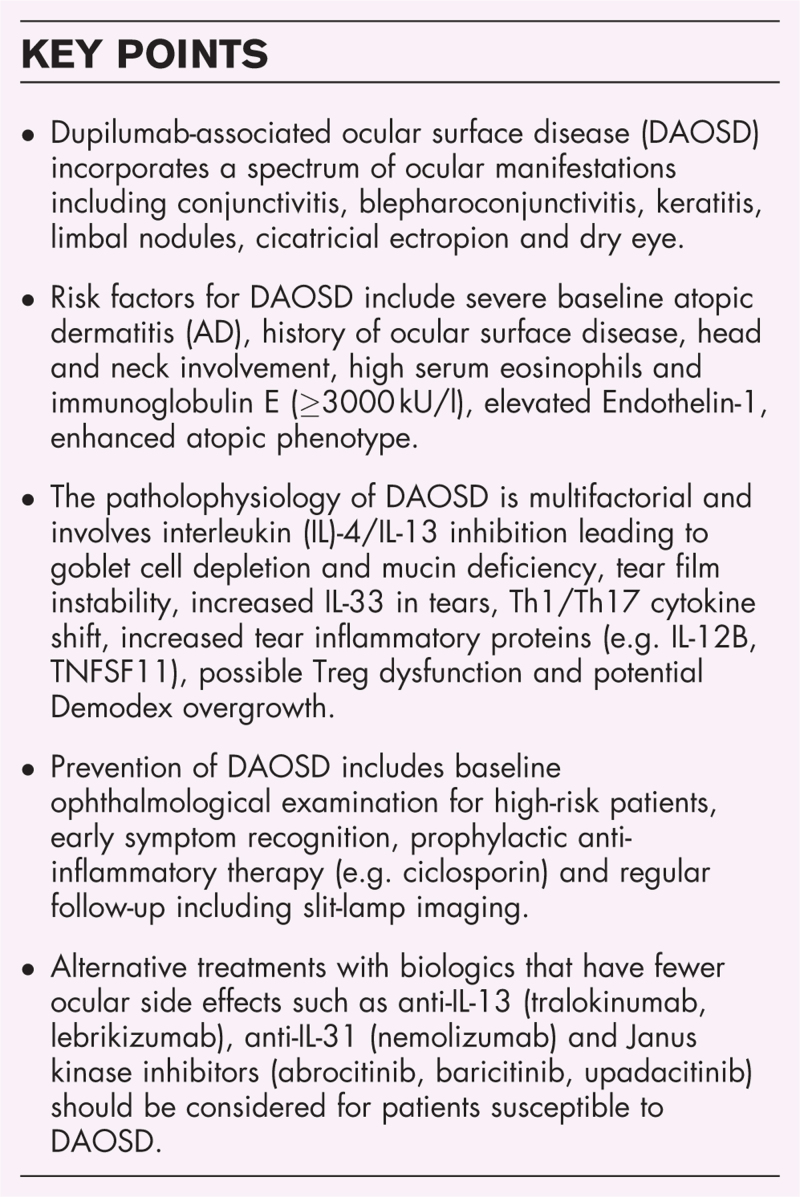
no caption available

## ATOPIC DERMATITIS

AD is a relatively common chronic allergic skin disease in which Th2 cytokine-mediated inflammation and impairment of the skin barrier are thought to be the key pathogenic events, with genetic associations and microbiome dysbiosis being involved [2]. Many AD patients (25–40%) develop an ocular surface condition, with allergic conjunctivitis being the most common form [3]. Among those with more severe AD, there is a higher risk of developing the most clinically severe form of allergic eye disease (AKC).

Previously topical immunosuppressives (corticosteroids, tacrolimus) were the mainstay of therapies, especially for younger AD patients. However, for adults with moderate-to-severe AD, more specific biologics have become available which are highly efficacious for many patients. In 2017, dupilumab was approved for treating moderate-to-severe adult cases of AD and, since 2022, Dupilumab has also been approved for children aged 6 months–5 years with moderate-to-severe AD.

## ATOPIC KERATOCONJUNCTIVITIS

AKC is an allergic eye disease which is either associated with atopic asthma or AD. In early studies of conjunctival tissue biopsies from patients with AKC or with another form of allergic eye disease – vernal keratoconjunctivitis (VKC), the immune cells in AKC were predominantly comprised of activated CD4^+^ T cells expressing Th1 cytokines whereas VKC involved Th2-T cells and eosinophils [1]. These cytokine profiles were also reflected in conjunctival tissue-derived polyclonal CD4-T cell lines [4], suggesting that AKC was involved both Th2 and Th1-cytokine pathways. Following these studies, a combination of topical ciclosporin A with dexamethasone was demonstrated to be clinically beneficial for AKC by inhibiting both Th1 and Th2 cells [5]. Subsequent studies have demonstrated that Th17 cells are also present [6].

Since the majority of AKC patients are usually receiving systemic immunosuppression for their associated chronic allergic disease (either AD or asthma), it was assumed that the inflammatory pathways were identical in the skin/lung and conjunctival tissues. However recent interesting findings discussed in this review suggest there is a divergence in the inflammatory disease pathways whereby, for some AD patients, the ocular inflammatory pathways are more complex, not solely due to Th2 cytokine-driven disease.

## DUPILUMAB-ASSOCIATED OCULAR SURFACE DISEASE: DEFINITION AND CLINICAL SPECTRUM

Dupilumab-associated ocular surface disease (DAOSD) is a term that encompasses a range of ocular manifestations occurring in patients treated with dupilumab. It is most commonly observed in individuals receiving treatment for moderate-to-severe AD. While conjunctivitis is the most frequently reported manifestation, DAOSD includes a spectrum of ocular surface presentations such as blepharoconjunctivitis, follicular conjunctivitis, limbal nodules, cicatricial ectropion, keratitis, and dry eye disease [7–14].

At the time of dupilumab's FDA approval in 2017, conjunctivitis was a known frequent adverse event. Higher rates of conjunctivitis were reported in patients treated with dupilumab compared to control groups in the pivotal Phase III clinical trials (8.6% in CHRONOS; 28% in LIBERTY AD CAFÉ [15,16]. Most cases were mild, with only one patient requiring discontinuation of dupilumab due to AKC. Notably, ophthalmologists were not involved in adjudicating these cases, and the increased incidence reported in LIBERTY AD CAFÉ was partly attributed to heightened awareness among investigators. Subsequent real-world data has revealed broader phenotypic manifestations and, in some cases, more severe ocular involvement. Retrospective cohort studies report incidences ranging from 32% to 43% [12,17], with isolated reports documenting rates as high as 70% [18].

## DUPILUMAB-ASSOCIATED OCULAR SURFACE DISEASE: CLINICAL FEATURES AND DIAGNOSIS

DAOSD typically presents within the first 16 weeks following initiation of dupilumab therapy. The incidence of new cases appears to plateau around 20–24 weeks and is uncommon beyond week 44 [19–21]. Symptoms include ocular irritation, itching, foreign body sensation, epiphora, and blurred vision. Clinical signs may consist of conjunctival hyperaemia, limbitis, meibomian gland dysfunction, punctate epithelial erosions, madarosis, symblepharon, and in advanced cases, ocular surface keratinisation or forniceal shortening (Table 1).

**Table 1:** Physical examination features of DAOSD

Mild	Meibomian gland dysfunction Conjunctival hyperaemia Papillary conjunctivitis Superficial punctate keratopathy
Moderate	Symblepheron formation Madarosis Forniceal shortening Limbitis
Severe	Ankyloblepharon Loss of fornices Ocular surface keratinisation

DAOSD, dupilumab-associated ocular surface disease.

Diagnosis is clinical and based on slit-lamp examination by an ophthalmologist. Key features to assess include the bulbar and tarsal conjunctiva, fornices, limbus, and cornea. Additional assessments may include tear film break-up time, fluorescein staining, and meibomian gland assessment. There are currently no laboratory or biomarker-based tests to definitively diagnose DAOSD.

## DUPILUMAB-ASSOCIATED OCULAR SURFACE DISEASE: EPIDEMIOLOGY AND RISK FACTORS

Interestingly, DAOSD appears predominantly in patients treated for AD. In contrast, patients receiving dupilumab for asthma, chronic rhinosinusitis with nasal polyps (CRSwNP), or eosinophilic oesophagitis show significantly lower rates of ocular adverse events [19,22–24]. The reason for this predilection remains unclear although, in some rhinosinusitis studies, nasal steroids were maintained which could have masked any DAOSD. Additionally, only a particular subset of AD patients appears to experience DAOSD. Identifying risk factors and potential biomarkers related to the development of DAOSD would enable clinicians to better risk stratify patients considering dupilumab treatment and take preventive measures for those most at risk, such as monitoring by an ophthalmologist.

Risk factors identified for DAOSD include a prior history of ocular surface disease and more severe baseline AD [17,25]. Other predictive factors include head and neck involvement, increased eosinophil counts, and an enhanced atopic phenotype [26,27].

Kido-Nakahara *et al.* examined several serum biomarkers, including LDH, eosinophils, immunoglobulin E (IgE), and Endothelin-1 (ET-1), in relation to the development of dupilumab-associated conjunctivitis and blepharitis [28^▪^]. While no statistically significant associations were confirmed, elevated ET-1 levels showed a trend toward association (*P* = 0.051), with higher ET-1 concentrations correlating with an increased likelihood of disease. It has previously been suggested that Th1 and Th17 responses may be associated with the pathology in DAOSD and blepharitis [29,30], and ET-1 has previously been reported to be associated with Th1 and Th17/22 responses. Therefore, dupilumab use in patients with high ET-1 levels may predispose them to an immune shift towards Th1 and Th17/22, contributing to the development of conjunctivitis and blepharitis. Additionally, ET-1 has been shown to suppress mucin secretion from goblet cells [31], potentially contributing to this subset of patients’ predisposition to DAOSD. In a recent paediatric AD study, elevated baseline serum IgE levels (≥3000 kU/L) were independently associated with DAOSD development and could be used as a predictive biomarker in children and young adults [32^▪^].

## DUPILUMAB-ASSOCIATED OCULAR SURFACE DISEASE: IMMUNOPATHOGENESIS

**FIGURE 1:**
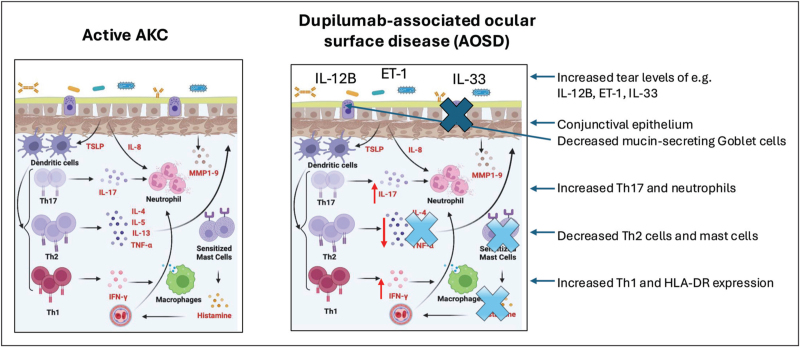
A summary of the cell and cytokine pathways known to be active at the ocular surface (tear fluids, conjunctival tissues) in active AKC (a) and DAOSD (b). AKC, atopic keratoconjunctivitis; DAOSD, dupilumab-associated ocular surface disease.

The precise mechanisms underlying DAOSD are not fully understood but are thought to be multifactorial (Fig. 1). Dupilumab inhibits the signalling of both IL-4 and IL-13 by binding to the IL-4Rα, which is expressed on conjunctival epithelial cells, fibroblasts, and corneal fibroblasts [33], implicating a direct role in modulating ocular surface Th2 responses. IL-4 and IL-13 are critical for maintaining conjunctival goblet cells and mucin secretion. Inhibition of these cytokines by dupilumab results in goblet cell depletion and MUC5AC deficiency, leading to tear film instability and inflammation [34,35]. A recent study in 7 of 39 AD patients receiving dupilumab who developed DAOSD at 16 weeks, identified a significant increase in tear fluid levels of IL-33 which was associated with decreased tear film breakup time, indicating dry eye [36]. Targeting costimulatory molecules expressed by several immune cells could offer an alternative treatment strategy for DAOSD where signalling pathways other than Th2 are involved, and recent mouse studies comparing CD30L (TNFSF8) and OX40 ligand (OX40L; TNFSF4) suggest CD30L might be an effective target for downregulating AD [37]. Whether such an approach could be effective for DAOSD or AKC remains unknown.

An alternative explanation for the development of DAOSD in a subset of AD patients is that, by inhibiting effector Th2-T cell responses with dupilumab, an imbalance in Th1/Th17/Th2 cytokines is created, allowing a shift towards a more Th1/17-driven response. One recent proteomics investigation used spectral flow cytometry to profile 92 proteins in tear fluids taken from AD patients (*n* = 37) before and after dupilumab treatment [38^▪▪^]. Comparing levels before and 8 weeks’ after dupilumab treatment, 31 inflammatory markers were significantly increased. Comparing 8 patients who developed DAOSD, there were 25 inflammatory proteins increased from baseline, including LAP TGFβ-1, TNFSF1, TNSF11, TNSF14, TNFβ, and IL-12B. After DAOSD onset in this cohort, there were further increases in IL-12B, CD5, CD8A, IL-8, LIF and IL-10RB, and a shift towards a Th1/17 profile. This comprehensive analysis of inflammatory markers in tear fluids supports the concept that dupilumab treatment leads to a shift in cytokine profiles in a subset of AD patients, and that tear fluids could be used to detect biomarkers to predict AD patients at risk of developing DAOSD. Furthermore, in a recent case study in which a patient with DAOSD switched from dupilumab to a JAKi (upadacitinib), this resulted in improvements of both dermatologic and ophthalmic signs, which correlated with an increase in conjunctival tissue expression of IL-4 and IL-13, and a decrease in HLA-DR, a marker for immune cell activation [39]. Such data suggest that in DAOSD, Th2 cytokines are acting as regulators of a more damaging Th1/17 mediated pathology. In DAOSD there have been reports of increased Demodex mite infestation, and a loss of conjunctival immune tolerance. It has been reported that IL-4/IL-13 knockout mice exhibited rapid colonization by Demodex mites, consistent with an impaired Th2 response [40].

The role of regulatory T cells (Treg) in tissue homeostasis has been the subject of extensive research in the last few years, with novel therapies involving Treg restoration being considered for several chronic inflammatory diseases. In AD, Treg are detected in the skin but have been shown to be functionally impaired and that has been suggested as a reason for the chronicity of AD [41]. In two recent randomized double-blind placebo-controlled phase 1b trials, a Treg-selective IL-2 receptor agonist (REZPEG) was administered to stimulate the Treg in 34 patients with moderate-to-severe AD [42]. This trial demonstrated clinical improvement at week 12, the treatment was well tolerated and all treatment-emergent adverse events were mild or moderate, with only 2 eye disorders recorded (5.9%), which is much lower than with dupilumab. Whether Treg impairment is also occurring in AKC, or in DAOSD remains unknown.

It has also been hypothesized that DAOSD may result from undertreatment or unmasking of previously undiagnosed AKC. Ocular surface diseases such as AKC are common in patients with AD (32–56%) [43–45], and can often go unnoticed before starting dupilumab therapy. Several studies report high rates of previously unrecognized blepharoconjunctivitis in AD patients initiating dupilumab. Maudinet *et al.* found that 64% of patients starting dupilumab had undiagnosed ocular surface inflammation at baseline, and pretreatment ophthalmology referral reduced the incidence of conjunctivitis from 25% to 13% [10].

Clinically, it is challenging to dissociate a blepharoconjunctivitis flare-up from DAOSD. Whether dupilumab simply increases the susceptibility to developing AKC in patients with existing disease or whether it is causing *de novo* pathology is unclear. However, histological evidence challenges the notion that DAOSD is simply an exacerbation of AKC. Bakker *et al.* found that conjunctival biopsies from dupilumab-treated patients lacked the characteristic eosinophilic infiltrate and other features typical of allergic conjunctivitis, suggesting a distinct pathologic entity [18]. In another recent omics-based study, research has identified metabolic and lipidomic differences between patients who develop DAOSD and those who do not, using ultrahigh-performance liquid chromatography and mass spectrometry [46^▪^].

Analysis of extracellular vesicles (EVs) and their miRNA cargo in tear film samples is also emerging as a potential diagnostic and pathophysiological tool within ocular surface diseases [47,48]. With advances in diagnostic capabilities, our understanding of why certain patients develop DAOSD will enable a more targeted approach to dupilumab therapy for atopic dermatitis.

## DUPILUMAB-ASSOCIATED OCULAR SURFACE DISEASE: MANAGEMENT

**FIGURE 2:**
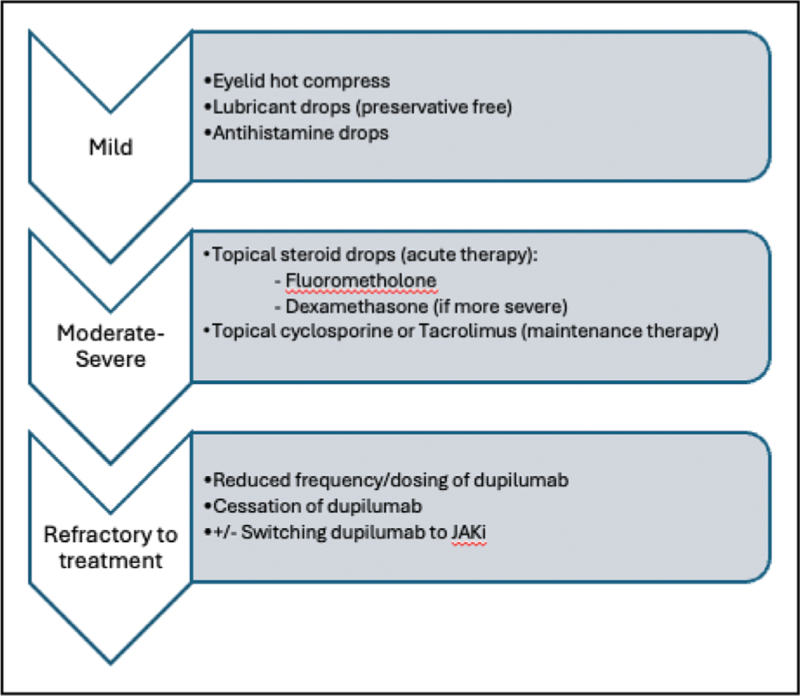
The management options available, depending on the clinical severity of the DAOSD. DAOSD, dupilumab-associated ocular surface disease.

Treatment of DAOSD depends on severity and is summarized in Fig. 2. Mild cases may respond to preservative-free lubricants and eyelid hygiene. Moderate to severe disease often requires topical corticosteroids or calcineurin inhibitors such as ciclosporin. Ciclosporin has demonstrated efficacy in increasing goblet cell density and restoring ocular surface immune balance.

In severe or refractory cases of DAOSD, discontinuation of dupilumab may be considered. The RESO-ADOC study [49^▪^], a multicentre observational study, reported that 30% of patients with DAOSD required cessation of dupilumab therapy, a substantially higher rate than that reported in clinical trials (<0.5%) [19–21]. The advent of multiple new alternative treatments for AD over the past 3 years, such as anti-IL-13 agents (Tralokinumab, Lebrikizumab [50^▪^]), anti-IL-31 (Nemolizumab [51]) and Janus kinase inhibitors (JAKi; abrocitinib, baricitinib, Upadacitinib [52]) may have reduced the threshold to stop dupilumab therapy compared to that in the clinical trials in 2016.

Importantly, the RESO-ADOC study showed that patients switched from dupilumab to a JAKi such as abrocitinib, baricitinib, or upadacitinib demonstrated rapid and sustained resolution of ocular symptoms (96% at follow-up visit 3; [49^▪^]). In contrast, only 45% of those who transitioned to tralokinumab experienced complete resolution, suggesting differential effects based on the therapeutic mechanism.

There is growing interest in the shared pathogenesis of dupilumab-induced ocular and extraocular adverse events, such as psoriatic dermatitis and arthritis, which have also shown responsiveness to JAKi [53]. Both presentations may share pathology in disrupted Th1/Th2 balance and their downstream effects.

## DUPILUMAB-ASSOCIATED OCULAR SURFACE DISEASE: PREVENTION

For patients with identified risk factors (e.g., severe AD, prior ocular surface disease), proactive management is key. Baseline ophthalmological examination and early intervention upon symptom onset may prevent progression to cicatrizing disease. Regular follow-up with slit-lamp photography can aid in documentation and monitoring. Eyelid hygiene and prophylactic topical anti-inflammatory therapy may also be considered in high-risk individuals.

Expert insights and unresolved questions:

(1) Are there predictive biomarkers for DAOSD?
(2) Should ophthalmic co-management be routine before dupilumab initiation in AD?
(3) Long-term ocular sequelae: rare cicatricial conjunctivitis – reversible or progressive?

## CONCLUSION

DAOSD is an increasingly recognized adverse event in patients with AD, characterized by goblet cell depletion, mucin deficiency, immune dysregulation, and tear film instability. While often mild and treatable, severe cases may result in vision-threatening complications. Dupilumab is highly effective for treating AD in moderate-to-severe cases, indicating that Th2-driven disease is involved. However, in patients with DAOSD, Th1/Th17 pathways are likely to be involved which will require broader-acting immunosuppressives.

As summarized in Table 2, recognition of risk factors, early ophthalmological evaluation, and targeted therapy can help mitigate its impact. Recent advances in molecular diagnostics may soon allow for improved risk stratification and personalized treatment approaches.

**Table 2:** Summary of dupilumab-associated ocular surface disease (DAOSD)

DAOSD	Key points
Terminology	Spectrum of ocular manifestations including conjunctivitis, blepharoconjunctivitis, keratitis, limbal nodules, cicatricial ectropion, dry eye; most cases occur within 16 weeks of dupilumab initiation; severity ranges from mild to sight-threatening; incidence plateaus by week 24
Risk factors	Severe baseline AD; history of ocular surface disease; head and neck involvement; high serum eosinophils and IgE (≥3000 kU/l); elevated Endothelin-1; enhanced atopic phenotype.
Immunopathogenesis	IL-4/IL-13 inhibition leads to goblet cell depletion and mucin deficiency; tear film instability; increased IL-33 in tears; Th1/Th17 cytokine shift; increased tear inflammatory proteins (e.g. IL-12B, TNFSF11); potential Demodex overgrowth; possible Treg dysfunction.
Management	Mild: eyelid hygiene, preservative-free lubricants, antihistamine drops. Moderate to severe: topical corticosteroids or ciclosporin. Severe or refractory: reduce frequency/dosing of dupilumab; discontinue dupilumab; switch to JAK inhibitors (e.g. upadacitinib, abrocitinib) showing >90% resolution in some studies
Prevention	Baseline ophthalmological examination for high-risk patients; early symptom recognition; prophylactic anti-inflammatory therapy (e.g. ciclosporin); regular follow-up including slit-lamp imaging
Alternative Treatments	Biologics with fewer ocular side effects: anti-IL-13 (tralokinumab, lebrikizumab), anti-IL-31 (nemolizumab), Janus kinase inhibitors (abrocitinib, baricitinib, upadacitinib).

AD, atopic dermatitis; IgE, immunoglobulin E; IL, interleukin.

Understanding the immunologic divergence between therapeutic success in AD and paradoxical inflammation on the ocular surface remains an important research priority.

## Acknowledgements

*The authors have no acknowledgements.*

### Financial support and sponsorship

*No funding was received for the publication of the manuscript. K.J. is the recipient of Moorfields Eye Charity funding.*

### Conflicts of interest

*Dr Calder has previously consulted for Allergan Inc, GSK plc, Santen and Yuyu Pharmaceuticals Inc. She is an Advisory Board member at Naegis Pharmaceuticals Inc.*

## REFERENCES AND RECOMMENDED READING

Papers of particular interest, published within the annual period of review, have been highlighted as:

▪ of special interest
▪▪ of outstanding interest
